# Gene expression endophenotypes: a novel approach for gene discovery in Alzheimer's disease

**DOI:** 10.1186/1750-1326-6-31

**Published:** 2011-05-14

**Authors:** Nilüfer Ertekin-Taner

**Affiliations:** 1 Mayo Clinic Florida, Departments of Neurology and Neuroscience, 4500 San Pablo Road, Birdsall 210, Jacksonville, Florida 32224 USA

## Abstract

Uncovering the underlying genetic component of any disease is key to the understanding of its pathophysiology and may open new avenues for development of therapeutic strategies and biomarkers. In the past several years, there has been an explosion of genome-wide association studies (GWAS) resulting in the discovery of novel candidate genes conferring risk for complex diseases, including neurodegenerative diseases. Despite this success, there still remains a substantial genetic component for many complex traits and conditions that is unexplained by the GWAS findings. Additionally, in many cases, the mechanism of action of the newly discovered disease risk variants is not inherently obvious. Furthermore, a genetic region with multiple genes may be identified via GWAS, making it difficult to discern the true disease risk gene. Several alternative approaches are proposed to overcome these potential shortcomings of GWAS, including the use of quantitative, biologically relevant phenotypes. Gene expression levels represent an important class of endophenotypes. Genetic linkage and association studies that utilize gene expression levels as endophenotypes determined that the expression levels of many genes are under genetic influence. This led to the postulate that there may exist many genetic variants that confer disease risk via modifying gene expression levels. Results from the handful of genetic studies which assess gene expression level endophenotypes in conjunction with disease risk suggest that this combined phenotype approach may both increase the power for gene discovery and lead to an enhanced understanding of their mode of action. This review summarizes the evidence in support of gene expression levels as promising endophenotypes in the discovery and characterization of novel candidate genes for complex diseases, which may also represent a novel approach in the genetic studies of Alzheimer's and other neurodegenerative diseases.

## Introduction

It is well established that the risk for many neurodegenerative diseases such as Alzheimer's disease (AD)[1,2], Parkinson's disease (PD)[2,3], frontotemporal dementia (FTD) and amyotrophic lateral sclerosis (ALS)[4,5] is under substantial genetic control. Identification of deterministic mutations with a Mendelian pattern of inheritance in familial forms of these diseases has led to significant progress in our understanding of their underlying pathophysiology. Nonetheless, such monogenic forms constitute only a fraction of these conditions (e.g. < 1% of all AD[1], 5-10% of all PD[2]); and risk for most neurodegenerative diseases, like other common diseases/traits, is thought to arise from multiple genetic variants and their interaction with each other, as well as environmental factors. Whereas investigation of large families with Mendelian inheritance patterns, using linkage followed by sequence analyses have generally proven useful in gene discoveries for monogenic forms of neurodegenerative diseases[1,5], association studies in unrelated case-control series have emerged as a more viable strategy in the common, multigenic and complex forms of these conditions[1-3]. The relative lack of success from candidate gene association studies coupled with technologic advances led to the emergence of genome-wide association studies (GWAS) as a promising approach in gene discovery for common, complex diseases/traits with heterogeneous and multigenic underpinnings.

The past several years have witnessed an explosion of GWAS that survey hundreds of thousands of genetic variants across the whole genome for association with disease risk in a hypothesis-independent manner. The recent widespread use of this approach became possible with the generation of a linkage disequilibrium (LD) map of common single nucleotide polymorphisms (SNPs) across the whole genome as part of the International HapMap Project[6,7]. This effort combined with the technological advances in high-throughput genotyping allowed for the production of chips that contain up to 1 million SNPs which provide information about most (but not all) of the common genetic variation (usually defined as having a minor allele frequency > 0.05) in the genome by tagging (marking) the un-genotyped SNPs through LD. GWAS emerged based on the underlying "common disease-common variant" hypothesis which posits that the risk for many common diseases such as coronary artery disease, diabetes mellitus and AD is conferred by multiple common variants [8]. This approach proved to be successful in the discovery of candidate risk variants or regions for many common diseases, including neurodegenerative diseases[1-3].

It became evident from these studies that although GWAS have identified numerous genetic loci for common diseases, they fell short of accounting for all of the genetic component of these conditions[8,9]. The reasons for this have been discussed in recent reviews[8,9] and include a) modest power of GWAS that utilize disease phenotype given relatively small effect sizes of risk variants, genetic heterogeneity of the disease (different disease risk variants at play in different subjects), and heterogeneity of the populations (different extent of LD); b) presence of rare disease risk variants or structural variants (such as insertions and deletions) that are not captured by the GWAS SNP platforms, c) presence of gene-gene and/or gene-environment interactions that are yet unexplored.

The SNPs identified through GWAS may reside near genes with no known or disease-relevant function. Some disease-associating variants may reside in LD regions harboring multiple genes, making it impossible to discern the true disease-risk gene. These represent relative weaknesses of GWAS resulting in part from the hypothesis-independent nature of this approach. On the contrary, this may also be perceived as a relative strength, since it may allow for the discovery of unexpected genes that would have been missed with a hypothesis-based approach. Nevertheless, the plethora of loci identified through GWAS need both functional confirmation and characterization, since their roles in disease risk are usually not inherently obvious.

Multiple different approaches are proposed to overcome these shortcomings of GWAS that are beyond the scope of this review. One of the proposed approaches involves the use of biologically relevant, quantitative phenotypes (endophenotypes) for discovery and characterization of disease risk genes[8,9]. This review focuses on gene expression levels as a potentially powerful group of endophenotypes and discusses the endophenotype concept, the evidence in support of a substantial genetic component for human gene expression, the GWAS examples that combine disease phenotype and expression endophenotypes, the use of gene expression endophenotype in the Alzheimer's and other neurodegenerative disease literature to-date and future directions.

## The Endophenotype Concept

The term "endophenotype" was first introduced in 1966 in the context of Drosophila genetics to contrast the phenotypes that are "obvious and external" (i.e. exophenotypes) with those that are "microscopic and internal" (i.e. endophenotypes)[10]. The term was applied in psychiatric genetics in the 1970s, specifically in schizophrenia genetics to describe molecular outcomes of gene activity, which lead to disease[11]. It was not until the 21^st ^century that this term reached more widespread usage, mainly in psychiatric genetics initially, but subsequently also in other complex conditions such as neurodegenerative[12,13], cardiovascular[14] and atopic [15] diseases. That the endophenotype approach was first advocated in psychiatric disorders partly stems from the need to have objectively quantifiable phenotypes associated with disease to avoid the imprecise and therefore heterogeneous nature of psychiatric diagnostic criteria[16], which is thought to contribute to the failure of genetic studies of psychiatric disorders. In a hypothetical construct that defines genotype-disease relationship, endophenotypes were proposed as quantitative phenotypes that are intermediate between genes and the disease outcome, that are directly influenced by a smaller number of genes than the disease phenotype, and that represent one of many facets of a disease[17]. Thus, another premise for this approach is the assumption that the underlying genetic architecture of the endophenotype will be simpler than that of the disease phenotype[17], though this may clearly not be the case[16]. Nevertheless, their closer proximity to gene action could lead to greater "genetic signal-to-noise" ratios[17,18], which may translate into larger effect sizes for the genetic variants under investigation, and thereby increased power to detect genetic loci of interest. Furthermore, given that endophenotypes are measurable traits regardless of disease state, their use can allow the inclusion of unaffected as well as affected subjects in genetic studies, which can also enhance power, especially in family-based studies[18,19].

Genes identified via the endophenotype approach may be more amenable to the study and discovery of genetic pathways underlying disease. This is partly due to the quantifiable nature of the endophenotype, which makes possible objective testing of the downstream effects of genetic perturbations, including development of animal models[20]. A potential caveat of the endophenotype approach is the fact that it represents only one of many aspects of the disease pathology. This may be a limitation for animal models that study the endophenotype rather than disease symptomatology as the outcome. That said, given the improbability of recapitulating the complete spectrum of a complex disease in an animal model and the potential difficulty in drawing parallels between animal and human symptomatology[20], a focused study of a "good" endophenotype could enhance our understanding of complex disease pathophysiology.

What constitutes a "good" endophenotype? A variety of criteria have previously been proposed to define "valid and useful" endophenotypes in a review, where a core set of "necessary and sufficient" criteria was identified[16]. Although a detailed discussion of these criteria is beyond the scope of this review, we will briefly mention those that are commonly proposed. Endophenotypes should be measured reliably and reproducibly upon multiple measurements of the same subject/sample. Given the proposition that endophenotypes are traits that can be used to map genetic variants underlying disease risk, heritability or having a measurable genetic component is a *sine qua non *of a useful endophenotype. Neuroimaging[18] and cognitive[17,21] endophenotypes are examples of heritable traits that are proposed for genetic studies of neuropsychiatric diseases. The underlying assumption is that genes influence the endophenotype in a way that is detectable prior to the clinical onset of disease. These changes in the endophenotype in turn leads to increased disease risk. Consequently, endophenotypes should be associated with the disease in the general population and also co-segregate with it within families. They should manifest changes that are detectable in the clinically-unaffected but at-risk subjects, such as family members of patients. We determined that plasma Aβ levels, which show variation in the general population, show significant elevations in cognitively-normal first-degree relatives of patients with late-onset AD[22] and are highly heritable traits[23]. Thus, we used plasma Aβ as an excellent endophenotype in a linkage study of late-onset AD pedigrees and mapped an AD risk locus on chromosome 10[19]. Endophenotypes need not, however, be disease-specific. For example, both neuroimaging and cognitive endophenotypes are quantitative traits that are variable and detectable in the general population. These endophenotypes may show changes that are associated with more than one psychiatric or neurologic disorder. Ideally, endophenotypes should be state-independent, and not change with the disease state or environmental factors. For example, although potentially useful endophenotypes for AD and possibly also other neurodegenerative diseases, both neuroimaging and cognitive endophenotypes are influenced by the disease state, as well as other variables such as age and gender[24]. It is therefore important to recognize and either statistically or technically control for such variables in genetic analysis of endophenotypes.

The evidence supporting a strong genetic component that influences human gene expression is discussed in the next section.

## Genetics of Human Gene Expression

In this section, 15 key studies using expression levels as an endophenotype to identify genetic loci or variants that influence human gene expression are reviewed (Tables 1 and 2). This approach is known as expression quantitative trait locus (eQTL) mapping via genetic linkage in families or association studies in unrelated populations. This section describes these studies highlighting their results which provide support for the existence of a substantial genetic component for gene expression in humans and how this component could be utilized to study the genetics of complex diseases and traits.

**Table 1:** Summary of studies on geneticsof human gene expression: Study characteristics

Reference	Reference ID	Organism	Tissue	Sample Size	Samples	Transcript Platform	Genotyping Platform
**Yan et al., 2002**	[28]	Human	Lymphoblastoid cell lines (LCL)	96	Subjects from the CEPH families (17-37 subjects who were heterozygouse for any given gene)	ABI Prism SNaPshot Multiplex Kit for 13 genes.	SNPs for 13 genes.

**Schadt et al., 2003**	[29]	Mouse	Liver	111	F2 mice constructed from two standard inbred strains, C57BL/6J and DBA/2J	23,574 transcripts (Rosetta Inpharmatics Merck).	>100 microsatellite markers.
		
		Z. mays (corn)	Ear leaf tissue	76	An F3 cross constructed from standard inbred lines of Z. mays	24,473 transcripts (Rosetta Inpharmatics Merck).	NA
		
		Human	Lymphoblastoid cell lines (LCL)	56	Subjects from four CEPH families	24,479 transcripts (Rosetta Inpharmatics Merck).	NA

**Cheung et al., 2003**	[30]	Human	Lymphoblastoid cell lines (LCL)	45	35 unrelated subjects from CEPH families vs. 1 reference pool of 10 subjects. 5 genes assessed in a larger sample size (49 unrelated CEPH subjects, 41 sibs from 5 CEPH families and 10 monozygotic twin pairs).	5000 random cDNA clones from IMAGE consortium.	Not done.

**Morley et al., 2004**	[31]	Human	Lymphoblastoid cell lines (LCL)	234	94 unrelated grandparents from CEPH families and ~140 subjects from 14 large CEPH families.	8,500 transcripts (Analysis restricted to 3554 most variable expression phenotypes).	2,756 autosomal SNP markers (SNP Consortium).

**Monks et al., 2004**	[32]	Human	Lymphoblastoid cell lines (LCL)	167 subjects	From 15 CEPH families	23,499 transcripts (25K human gene oligonucleotide microarray)	346 autosomal genetic markers.

**Cheung et al., 2005**	[33]	Human	Lymphoblastoid cell lines (LCL)	57	Unrelated CEPH subjects.	374 transcripts (subset of Morley et al. 2004); Affymetrix Human Genome Focus arrays.	770,394 SNPs.

**Stranger et al., 2005**	[34]	Human	Lymphoblastoid cell lines (LCL)	60	HapMap Unrelated Caucasian Subjects (CEU)	1,433 transcripts for 630 genes (Illumina BeadArray, custom).	753,712 SNPs

**Stranger et al., 2007 (Nature Genetics)**	[35]	Human	Lymphoblastoid cell lines (LCL)	270	HapMap Subjects, 4 populations (30 Caucasian trios (CEU), 45 unrelated Chinese (CHB), 45 unrelated Japanese (JPT), and 30 Yoruba trios (YRI)).	14,456 transcripts for 13,643 genes (Illumina, Sentrix Human Whole Genome-6 Expression BeadChip version 1).	>2.2 million SNPs per population.

**Stranger et al., 2007 (Science)**	[36]	Human	Lymphoblastoid cell lines (LCL)	210	HapMap unrelated subjects, 4 populations (60 Caucasians (CEU), 45 Chinese (CHB), 45 Japanese (JPT), 60 Yoruban (YRI)).	14,925 transcripts for 14,072 genes	Phase 1 HapMap SNPs and CNV data from comparative genomic hybridization (CGH) array of 26,574 clones.

**Dixon et al., 2007**	[37]	Human	Lymphoblastoid cell lines (LCL)	400	Affected and unaffected children from families with an asthma proband.	54,675 transcripts representing 20,599 genes (Affymetrix HG-U133 Plus 2.0 chip).	109,157 SNPs for 830 subjects (Illumina Sentrix Human-1 Genotyping BeadChip) and 299,116 SNPs for 378 subjects (Illumina Sentrix HumanHap300 BeadChip).

**Goring et al., 2007**	[38]	Human	Lymphocytes (not transformed cells)	1240	Multigenerational Mexican-American families from the San Antonio Family Heart Study (SAFHS).	19,648 transcripts for 18,519 genes (Illumina, Sentrix Human Whole Genome-6 Expression BeadChip version 1).	432 highly polymorphic microsatellite markers.

**Emilsson et al., 2008**	[39]	Human	Blood and adipose tissue	1,002 blood and 673 adipose cohorts.	Icelandic Family Blood (IFB) cohort (N = 1,002) and the Icelandic Family Adipose (IFA) cohort (N = 673).	20,877 transcripts	1,732 microsatellite markers for linkage analysis and 317,503 SNPs for association analysis (150 unrelated subjects).

**Schadt et al., 2008**	[40]	Human	Liver	427	Unrelated subjects.	39,280 transcripts for 34,266 genes (Custom Agilent microarray).	782,476 unique SNPs (Affymetrix 500 K and Illumina 650Y)

**Myers et al., 2007**	[41]	Human	Brain	193	Human brain samples that are neuropathologically normal.	14,078 transcripts (Illumina HumanRefseq-8 Expression BeadChip).	366,140 SNPs (Affymetrix 500 K).

**Webster et al., 2009**	[42]	Human	Brain	176 cases and 188 controls.	Human brain samples that are neuropathologically normal and with pathological Alzheimer's disease (AD).	8,650 transcripts (Illumina HumanRefseq-8 Expression BeadChip).	380,157 SNPs (Affymetrix 500 K).

**Table 2:** Summary of studies on geneticsof human gene expression: Analyses, results, conclusions.

Reference	Reference ID	Analytic Approach	Results	Conclusion
**Yan et al., 2002**	[28]	Comparison of relative allelic expression levels within the same cellular sample.	Significant differences in allele-specific expression observed for 6 of 13 genes. Mendelian inheritance detected for expression levels, inherited together with genetic markers.	Gene expression levels can be used to detect genetics of disease susceptibility.

**Schadt et al., 2003**	[29]	eQTL (expression quantitative trait locus) linkage analysis.	Differential expression detected for 7,861 of 23,574 (>33%) genes in the parental and ≥10% genes in the F2 strain. 9-16% genes have eQTLs with LOD scores > 4.3. Gene expression profiling identified three distinct expression patterns for 280 genes that distinguish mice at the lower 25th percentile of an obesity trait (fat-pad mass = FBM) and two groups at the upper 25th percentile of the FPM trait. These 280 genes were enriched for eQTLs. Linkage analysis of the obseity trait focused on groups with distinct expression patterns improved the signal.	Gene expression levels can be used to identify more refined disease sub-groups, genes and pathways that are implicated in the disease phenotype. These have implications in understanding genetics of complex diseases and drug discovery aimed at more homogeneous sub-groups of distinct expression patterns.
			
		eQTL (expression quantitative trait locus) linkage analysis.	18,805 (77%) genes with differential expression. Of these, 6,481 genes with ≥ 1 eQTL with LOD score > 3.0. Total of 7,322 eQTLs. Interactions detected in <10% of eQTL.	
			
		Variance components analysis to test heritability.	2,726 genes with differential expression (11%). Of those 29% have a detectable heritability.	

**Cheung et al., 2003**	[30]	Utilized 3-4 replicate measurements per person. Calculated variance ratio of each gene expression by dividing the variance of expression levels among subjects by that within subjects (using replicates).	50% of genes on the arrays are expressed in the LCLs. 813 genes with valid observations had variance ratios of 0.4-64. 5 genes evaluated in larger group and found to have highest variance among unrelateds, then sibs then monozygotic twins (10 pairs).	There is natural variation to gene expression levels which is at least in part determined genetically. Genetic differences among individuals may account for variations in gene expression and suggest underlying heritability.

**Morley et al., 2004**	[31]	Variance of expression levels detected from 94 unrelated subjects. 3554 expression phenotypes with greater variation between subjects than within subjects (replicates) were used for further analysis. Genome-wide linkage analysis conducted for these phenotypes in 14 CEPH families.	Found 984 expression phenotypes with pointwise linkage p < 0.05 genome-wide, more than the 178 false positives expected by chance alone. 142 phenotypes have pointwise p < 0.001, which exceeds chance (3.5 false positives expected). Of the top 142, 27 have a cis-(within 5 Mb) and 110 have a trans-regulator. Of the 984, 164 have multiple regulators (152 with multiple trans and both cis+trans for 12). There are linkage regions with multiple expressions linking to it, called "hotspots". Genes that map to one hotspot have expression levels with higher than expected correlations. Some of these genes have close physical locations. Some cis-SNPs show differential allelic expression.	Genetic factors that influence variation in human gene expression can act in cis (5 Mb) or trans. There are transcriptional "hotspots", which may contain "master regulators" of multiple genes. Mapping genetic factors that influence gene expression could help with the understanding of human biology and disease.

**Monks et al., 2004**	[32]	Variance components analysis to test heritability and eQTL analysis. Comparison of biological pathways within GO and KEGG, using genes clustered by genetic correlations (GC) and Pearson's correlations (PC). Correction for multiple testing with Bonferroni and false discovery rate (FDR).	2,430 of 23,499 genes differentially expressed in ≥50% of children. Of these, 762 were heritable with FDR of 0.05 and median heritability of 0.34. These genes were enriched for immunity-pathways. 22 genes have significant eQTLs at genome-wide level. Did not detect "hotspots". 574 genes analyzed for GC and PC showed that both clusters have similar pathway coherence for GO, but GC has better pathway coherence for KEGG pathways.	Genetic factors influence gene expression in LCL. Important to test other tissues. Random samples may not have transcriptional "hotspots". Gene expression genetics may identify novel biological pathways.

**Cheung et al., 2005**	[33]	Follow-up association study for the significant linkage findings from Morley et al. (2004). Linear regression association for 374 expression levels with prior evidence of *cis*-linkage, using SNPs near linkage peaks (± 50 kb). Expression GWAS (eGWAS) for 27 top *cis*-linkage phenotypes, using >770,000 SNPs. Comparison of prior linkage and association results.	65 of 374 expression levels have ≥1 SNP that associates at p < 0.001, 12 with p < 1E-10 and 133 with p < 0.01. Same proportions of associations found for the 5', 3' and genic regions. 14 out of top 27 *cis*-linkage regions showed genome-wide associations. 12 of those were *cis *only, 1 was *cis*+*trans *and 1 was *trans *only. One gene with strong cis-linkage and association was submitted to two functional assays which confirmed presence of a functional variant that influenced gene expression by modifying strength of RNA polymerase II binding.	Strong linkage predicts strong association for expression levels. eSNPs NOT enriched for 5' or 3' end. eGWAS is feasible and may lead to genetic determinants of expression phenotypes.

**Stranger et al., 2005**	[34]	Analyzed 374 of 630 genes with expression signals above background and most variable. Linear regression association for these genes (688 probes in total). Three methods for multiple-test correction: Bonferroni, FDR and permutations.	Good concordance between the 3 multiple test correction methods. For 10-40 of 374 genes, *cis*-SNPs (1 Mb from genomic midpoint of gene) are detected at genome-wide level by ≥1 statistical method. Only 3 *trans *hits were observed which are more likely to be false positive.	eGWAS can identify variants with regulatory activity

**Stranger et al., 2007 (Nature Genetics)**	[35]	Linear regression association analysis in 4 ethnic populations. Heritability estimates in Caucasian and Yoruba trios. Tested for significance by 10,000 permutations and FDR. Candidate trans-SNP analysis (SNPs with *cis*-effects, non-synonymous, splicing, microRNA SNPs).	10% (4,829) and 13% (6,482) of all probes analyzed has heritability >0.2 in CEU and YRI trios, respectively, with 958 overlapping genes. 154 CEU and 217 YRI genes have heritability >0.5, with overlap of 9 genes. 831 genes with significant *cis *in at least 1 population; 310 in at least 2; 62 in all four. Most detected genes have heritability estimates above 0.2. Pooling populations captures some additional genes with smaller effect size. Most *cis*-associations are in genic and immediate intergenic regions. 108 genes with significant *trans *association in ≥1 population, 16 genes in ≥2 and 5 in all 4 populations. Most *trans*-SNPs also have *cis*-effects. CEU population had most divergent expression profile from other populations, likely due to age of cell lines. There were 60 cell lines that were measured on 2 different arrays, which had high correlations in overlapping results, suggesting transcript measurements are stable across different experiments, measurement times and platforms.	There is a substantial number of heritable expression traits detectable in small population (30 trios), but also substantial non-genetic variation. Substantial overlap between different ethnic groups for significant eSNPs. Ethnic differences could in part be due to differences in SNP frequencies. Most eSNPs act in-*cis*.

**Stranger et al., 2007 (Science)**	[36]	Linear regression association analysis in 4 ethnic populations. *Cis*-SNPs defined as 1 Mb from midpoint of probe and *cis*-CNVs within 2 Mb. Permutation-based p values ≤ 0.001 deemed significant. Overlap with Nature Genetics study unclear.	Of 14,072 genes, 888 have ≥1 SNPs with significant association in ≥1 ethnic group, 331 of which were significant in ≥2 ethnic groups and 67 of which in all 4 populations. Of 14,072 genes, 238 have ≥1 CGH clone with significant association in ≥1 ethnic group, 28 of which were significant in ≥2 ethnic groups and 5 of which in all 4 populations. Not all CGH clones have detectable CNVs. 1322 CNV clones detected. 99 genes associate with ≥1 CNV clone, in ≥1 ethnic group, 34 of which with ≥2 ethnic groups, and 7 in all 4 populations. Most CNV associations cannot be detected by SNPs (87%).	Both SNP and CNV associations replicate across ethnic groups. CNVs appear to exert their effects by disrupting both regulatory regions as well as the genic regions. Survey of structural variants in addition to SNPs is important in eGWAS.

**Dixon et al., 2007**	[37]	Variance components analysis to test heritability and eQTL analysis on the subset of transcripts with heritabilities > 0.3. Multiple testing corrections by FDR.	No significant differences between asthmatics and non-asthmatics (unchallenged cells). 15,084 transcripts (28%) = 6,660 genes have heritabilities > 0.3. Traits with higher heritability have SNPs that explain a bigger percentage of their heritability and therefore also have a larger lod score of association (on average peak SNP explains 18.2% heritability). SNP interactions could explain transcript levels not explained by single SNPs. *Trans *effects weaker than *cis*. Highly heritable traits enriched for chaperonins, heat shock proteins, cell cycle progression, RNA processing, DNA repair, immune response.	Joint analysis of disease GWAS and eGWAS identified potential candidate genes for asthma (ORMDL3), Crohn's disease (PTGER4), NIDDM (PHACS), thalassemia (HBS1L). eGWAS is a useful approach to detect disease SNPs with a functional role.

**Goring et al., 2007**	[38]	Variance components analysis to test heritability and eQTL analysis. *Cis-*QTL = multipoint lod score at the location nearest the underlying structural gene. *Trans*-QTL = located on a different chromosome than its transcript. Multiple testing correction by FDR. Tested HDL-C concentrations for correlations with cis-regulated transcripts to identify *cis*-SNPs that also influence HDL-C.	16,678 transcripts (84.9%) were heritable with median heritability estimate of 22.5%. RefSeq transcripts have higher heritability estimates than non-RefSeq transcripts. At an FDR of 5%, identified 1,345 *cis*-regulated transcripts (6.8%) with median effect size of 24.6%. More significant *cis*- than *trans*-QTLs. Strongest QTLs tend to be *cis*. Identified a functional *cis*-SNP in VNN1 that associate with its expression and HDL-C levels.	Lymphocytes may provide more accurate representation of natural gene expression state than lymphoblasts, though there is overlap. *Cis*-regulation more stable across studies, tissues and stronger. No evidence of master regulators in this study.

**Emilsson et al., 2008**	[39]	Correlations between obesity traits and blood and adipose tissue expression levels. Variance components analysis to test heritability and eQTL analysis. Linear regression association analysis. Multiple testing correction by FDR. Generated connectivity matrix of genes with high correlation of expression in adipose tissue, compared human and mouse data, identified GO categories enriched for co-regulated genes.	Adipose tissue expression levels (63-72%) correlate better with obesity traits than do blood expression levels (3-9%). 55% of blood and 75% of adipose tissue transcripts are significantly heritable, with average heritability of 30%. 2,529 (12%) significant *cis*-eQTLs in blood, and 1,489 (7%) in adipose tissue. >50% of significant adipose tissue *cis*-eQTLs also significant in blood. Traits with higher heritability of greater reproducibility. Much less significant *trans*-eQTLs. No evidence of master-regulators. 2,714 (12.9%) significant *cis*-SNPs in blood and 3,364 (16%) in adipose tissue. Identified genes that are correlated in both human and mouse adipose tissue and enriched in macrophage activation pathways. cis-eSNPs for the expression traits in this network also influence obesity traits.	Significant overlap in genetic factors underlying gene expression in two different tissue types, but expression levels from clinically-relevant tissue correlates better with clinical-phenotypes. Expression correlation networks combined with *cis*-eSNPs could potentially identify genes/pathways underlying complex clinical phenotypes.

**Schadt et al., 2008**	[40]	Linear regression association analysis. *Cis *eQTL defined as being 1 Mb from transcription start or stop site of the gene. Multiple testing corrected by Bonferroni or FDR approaches. Compared significant eQTL results to those from published disease GWAS for Type 1 diabetes and coronary artery disease.	At Bonferroni adjusted p < 0.05, 1,350 expression traits (1,273 genes); at FDR <10%, 3,210 traits (3,043 genes) identified to have at least one significant *cis *eSNP, which explain 2-90% expression variation. Of the blood and adipose expression traits present on the liver expression microarrays, 30% had *cis *eQTLs that overlapped with the 3,210 significant liver *cis *eQTLs. *Trans *eQTLs significant at Bonferroni p < 0.05 were 242 traits (236 genes), and at FDR <10% were 491 traits (474 genes). Identified *SORT1 *and *CELSR2 *as candidate genes for coronary artery disease and LDL cholesterol levels, and *RPS26 *for Type 1 diabetes.	Evidence of common genetic control between tissues as well as tissue-specific genetic control of expression. Significant *trans *eQTLs only a fraction (15%) of *cis *eQTLs. Increase in sample size bigger impact on power than increasing genetic coverage by SNPs. *Cis *eQTLs combined with expression networks in humans and rodents and known biological pathways (such as KEGG) may help identify disease-susceptibility genes in regions of LD. Not all disease-SNPs will be eSNPs but strong expression association for disease-SNPs provides additional confidence for the candidates.

**Myers et al., 2007**	[41]	Linear regression association analysis. *Cis *eSNPs defined as being within the gene or 1 Mb its 3' or 5' end. Multiple testing corrected by permutation approaches.	58% of the transcriptome has expression in ≥5% of control brains. Of these 21% correlate with a *cis *or *trans *eSNP. 433 significant *cis *eSNPs (99 transcripts), and 16,701 significant *trans *eSNPs (2,876 transcripts). Enrichment of significant *cis *vs. *trans *associations maximized within ~70 kb of transcripts. MAPT *cis e*SNPs with alleles on the major haplotype (H1) are associated with higher transcript levels. Few common results with lymphoblast eGWAS.	Evidence for genetic control of human brain gene expression. Brain eSNPs may be used in conjunction with disease-SNPs for neurologic or psychiatric illnesses to identify functional variants.

**Webster et al., 2009**	[42]	Linear regression association analysis. Cis eSNPs defined as being within the gene or 1 Mb of its 3' or 5' end. Analyzed cases and controls both separately and jointly. In the combined analysis, tested for diagnosis effects on expression by comparing model with diagnosis only vs. one with diagnosis, SNP and diagnosis × SNP interaction. Multiple testing corrected by permutation approaches. Network analysis was done on the transcripts with a significant eQTL (p ≤ 0.01) and those without a significant eQTL but were differentially expressed between ADs and controls.	58% of the transcriptome has expression in ≥5% of AD brains. Hybridization date and APOE had strongest influence and post-mortem interval least influence on brain expression levels. 1,829 significant *cis *eSNPs in the combined sample and 656 *trans *eSNPs. 27% of all eQTLs with significant interaction term with diagnosis. 37% of *cis *eSNPs that interact with diagnosis overlap with those found in just the control brains (Myers et al). 18% overlap between previous report and *cis*+*trans *effects without diagnosis interaction and 9% overlap for those with interaction. Effect size of eSNPs that are closer to the transcription start sites are larger. Identified clusters of transcripts that are enriched for certain ontology groups and that contain "hub" genes with expression levels that correlate with many other transcripts.	Transcriptome measurements in disease-relevant tissue is important. Brain transcriptome appears to be unique. eQTLs may be used as biomarkers for classifying preclinical subgroups. eQTL approach may help distinguish true disease risk variants. Using tissue from subjects with disease may be needed to capture most eSNPs that have disease interactions, though significant eSNPs without disease interactions and some with disease interactions can be identified in control and disease tissue equally well.

Genetic linkage analysis of whole transcriptome expression levels in yeast determined that 1,528 of the 6,215 genes tested had natural variation in their expression levels and 570 of these gene expression phenotypes showed linkage to ≥ 1 loci in the yeast genome[25]. This study demonstrated that genetic factors account for a substantial proportion of variation in gene expression levels, even in a single cell organism. The first study in humans to evaluate genetics of gene expression (also known as the genetical genomics approach[26]) utilized lymphoblastoid cell lines (LCL) from the CEPH[27] (Centre d'Etude du polymorphisme humain) repository of Caucasian, multi-generational families[28]. Yan et al. screened 96 subjects and identified 17-37 individuals who were heterozygous for a SNP in 13 target genes. Measuring the mRNA of these 13 genes in the same cellular sample, they assessed relative expression of the two alleles of the SNPs and identified evidence of allele-specific expression for 6 of 13 genes in a subset of their samples. Examining the gene expression levels as a phenotype, they identified evidence of Mendelian inheritance for two expression phenotypes that co-segregated with nearby genetic markers in two families. These results provided the foundation to utilize the "genetical genomics" approach in a more high-throughput and systematic fashion.

Schadt et al. provided a comparative analysis of gene expression genetics in mouse, Zea mays and human, in a pioneering study which showed the generalizability of this approach to different organisms[29]. In this study, an eQTL linkage analysis was performed using 23,574 transcript expression levels measured in livers of 111 mice from the F2 generation of two standard inbred strains, C57BL/6J and DBA/2J and >100 microsatellite markers. Differential gene expression was detected for >33% of the genes in the parental and ≥10% genes in the F2 strain. 9-16% genes have eQTLs with LOD scores > 4.3. To identify transcripts that influence the complex obesity trait in these mice, the authors compared the gene expression profiles of mice at the lower vs. upper 25^th ^percentile of an obesity trait and identified three distinct expression patterns for 280 genes. Importantly, these 280 genes were enriched for eQTLs and linkage analysis of the obesity trait performed on the subgroups with distinct expression patterns improved the linkage signal. These results established a paradigm for the combined use of gene expression traits and another complex trait of clinical relevance for improved mapping of genetic factors that influence the complex clinical trait by affecting gene expression levels.

Analysis of corn (*Z. mays*) ear leaf tissue using the eQTL approach in the same study[29] identified that 77% of genes had differential expression in this organism, 26% had ≥ 1 eQTL with LOD score > 3.0 and there appeared to be genetic interactions between some of the eQTLs. Schadt et al. also studied a small number of human LCLs of 56 subjects from four CEPH families by variance components analysis and identified differential expression for 11% of the genes assayed of which about a third had detectable heritability. Overall, these findings demonstrate the complexity of gene expression genetics ranging from a "simpler" plant organism to humans, but also the applicability of genetical genomics approach in tracking eQTLs in different organisms.

One of the first studies which established the natural variation of gene expression levels in humans, evaluated LCLs of 35 unrelated subjects from CEPH families vs. 1 reference pool of 10 subjects[30] utilizing 3-4 replicate measurements per person. They determined that for many genes (n = 813) between-person gene expression variations were higher than within-person variations, which are due to technical variability. Evaluation of 5 genes, which revealed highest variance among unrelateds, then sibs then monozygotic twins, provided proof of principle for a genetic component underlying at least some of the variability in human gene expression.

Seven of the studies[31-37] following these initial reports utilized human LCL for eQTL mapping. Three of these studies[31-33] assessed cell lines from the CEPH repository[27], similar to the prior reports[28-30], three utilized LCL from the HapMap consortium[6,34-36] and one studied samples from families with an asthma proband[37]. Morley et al.[31] measured levels of 8,500 transcripts in 94 unrelated subjects from CEPH families and identified 3,554 (42%) transcripts with greater between-subject (biological) variation than within-subject (technical). Genome-wide linkage analysis of these 3,554 expression phenotypes in 14 CEPH families (n~140) detected evidence of significant linkage for 984 (28%) transcripts with genome-wide p < 0.05 and for 142 (~4%) with genome-wide p < 0.001, which far exceeds the numbers expected by chance alone. When these eQTLs were distinguished as *cis*- or *trans*-regulators, defined in this study as linkage regions within or outside 5 Megabases (5 Mb) of the target gene, respectively, most of the top 142 eQTLs were found to be *trans*-regulators. Some expression traits had multiple significant eQTLs. There were linkage regions with multiple expressions linking to it, termed as expression "hotspots" or "master regulators". Importantly, genes that map to one hotspot had expression levels with higher than expected correlations and some of them had close physical locations.

Monks et al.[32], also assessed LCLs from CEPH families (n = 167), though they measured a larger number of transcripts than Morley et al.[31], with 23,499 expression phenotypes that were evaluated by variance components analysis for their heritability, as well as eQTL analysis. They determined that 10% of the genes were differentially expressed in ≥50% of children, and a third of these were heritable at a false discovery rate (FDR) of 0.05 and median heritability of 0.34. The heritable transcripts were enriched for immunity pathways. Twenty-two genes had significant eQTLs at genome-wide level, eight of which were within 5 Mb of the gene (i.e. *cis*-regulating). In contrast to Morley et al.[31], Monks et al.[32] did not find an enrichment for transcription "hotspots" over what would be expected by chance alone, based on simulation studies. The authors studied a subset of 574 transcripts with heritability for pairwise genetic correlations (GC) and Pearson's correlations (PC), which does not account for a genetic component, followed by pathway analysis using the Gene Ontology (GO; http://www.geneontology.org/) and Kyoto Encyclopedia of Genes and Genomes (KEGG; http://www.genome.jp/kegg/) databases. This analysis revealed that genes clustered by both GC and PC had similar pathway coherence for GO, but that GC gene clusters had better pathway coherence for KEGG pathways. This suggests that analysis of pairwise genetic correlations between gene expression phenotypes may identify novel biological pathways that may not be possible by approaches which neglect the genetic component of correlations.

In a follow up to their prior linkage study[31], Cheung et al.[33] assessed 57 unrelated CEPH subjects in an association study for 374 expression phenotypes with prior evidence of linkage at p < 0.02 and *cis*-SNPs near linkage peaks (± 50 kb of target gene). Additionally, they evaluated the top 27 *cis*-linkage expression phenotypes (p < 3.7 × 10^-5^), in a GWAS using >770,000 SNPs. They determined that 65 of 374 expression levels have ≥1 SNP that associates at nominal p < 0.001, 12 with p < 10^-10 ^and 133 with p < 0.01. Same proportions of associations were found for the 5', 3' and genic regions. Fourteen out of the top 27 *cis*-linkage regions showed associations significant at the genome-wide level after Bonferroni corrections for the number of SNPs tested. Twelve of those top 14 associations were *cis *only, one was *cis*+*trans *and one was *trans *only. The authors also performed functional analysis for one of the top genes and confirmed the presence of a functional variant that influenced gene expression by modifying strength of RNA polymerase II binding. This study demonstrated that strong linkage also predicts strong association for expression levels and expression GWAS (eGWAS) may be a feasible and powerful approach to identify genetic determinants of expression phenotypes.

In the first of three studies[34-36] performed using HapMap LCL, Stranger et al.[34] analyzed 60 samples for levels of 1,433 transcripts (630 genes), then performed eGWAS for 688 transcripts (374 genes) with highly variable expression signals above the background. They compared and identified good concordance between three methods for multiple corrections: Bonferroni, FDR and permutations. There were 10-40 genes which had *cis*-SNPs (defined as 1 Mb from genomic midpoint of gene) significant at genome-wide level by ≥1 statistical method, whereas *trans *signals were detected only for 3 genes.

In a subsequent larger study, Stranger et al.[35], investigated LCL from four HapMap populations: 30 Caucasian trios (CEU), 30 Yoruba trios (YRI), 45 unrelated Chinese (CHB) and 45 unrelated Japanese (JPT). Analysis of 14,456 transcripts (13,643 genes) revealed a genetic component with heritabilities > 0.2 in 10% (4,829) and 13% (6,482) of the transcript probes analyzed in the CEU and YRI trios, respectively, with 958 overlapping genes. There were 154 CEU and 217 YRI genes with substantial heritability >0.5, with overlap of 9 genes. There were 831 genes (6% genes tested) with a *cis*-association significant in at least 1 population at p < 0.001 after 10,000 permutations; 310 in at least 2 and 62 in all four populations. There were many less *trans*-associations, with 108 significant genes in ≥1 population, 16 genes in ≥2 and 5 in all 4 populations. These findings demonstrate the presence of both overlap and diversity in significant eSNPs across different ethnic groups. More than 50% of the significant eQTLs had heritability estimates > 0.2, though pooling populations to increase sample size captured some additional genes with smaller effect sizes. Most *trans*-SNPs were also found to have *cis*-effects and most *cis*-associations were in genic and immediate intergenic regions, suggesting that *cis*-variants may be more abundant and/or may have stronger effects than *trans*-variants. This study also evaluated the influence of technical variability on gene expression measurements by testing 60 cell lines on two different arrays, that led to results with high correlations, suggesting that transcript measurements are stable across different experiments, measurement times and platforms. They also identified that CEU population, which had the most aged cell lines, also had the most divergent expression profile from other populations, drawing attention to the potential technical concerns with LCL.

To investigate the contribution of a different type of genetic variation, namely copy number variants (CNVs) to gene expression levels, Stranger et al.[36], performed association of 14,925 transcripts (14,072 genes) with CNVs in the same four HapMap populations[35] and determined that there are significant CNV associations that replicate across ethnic groups as well as those that are unique to one ethnic group. Mapping of CNVs vis à vis the genes suggested that CNVs exert their effects by disrupting both regulatory regions as well as the genic regions and altering gene dosage. Notably, most CNV associations cannot be detected by SNPs (87%), indicating the importance of the survey of structural variants in addition to SNPs in eGWAS.

In a study of 400 LCL from affected and unaffected children of families with an asthma proband, Dixon et al.[37], studied 54,675 transcripts (20,599 genes). First, there were no significant differences in the expression profiles between asthmatics and non-asthmatics (unchallenged cells). Twenty-eight percent of the transcripts (15,084) corresponding to 6,660 genes have heritabilities > 0.3, which is in good agreement with prior reports[29,32]. 1,989 transcripts had significant associations at genome-wide level, where many of the strongest associations were in *cis*. This study did not identify master regulators with strong effects. Combined investigation of these eGWAS results with those of published GWAS on some diseases, identified potential candidate genes for asthma, Crohn's disease, diabetes and thalassemia implying that eGWAS is a useful approach to detect disease SNPs with a functional role.

In one of the largest eQTL studies to date, Goring et al.[38] analyzed 1240 lymphocyte (not LCL) samples collected from multigenerational Mexican-American families from the San Antonio Family Heart Study (SAFHS) for 19,648 transcripts (18,519 genes). Nearly 85% of the transcripts (16,678) were significantly heritable with a median heritability estimate of 22.5%. This estimate is similar to those from some LCL studies[29,32,37]. There were 1,345 significant *cis*-regulated transcripts with median effect size of 24.6%. There were many less *trans*-regulators, again reinforcing the idea that *trans *effects likely have smaller effect sizes than those in *cis*. This study was able to replicate the *cis*-findings of Morley et al.[31], but not those in *trans*, which suggests higher stability of *cis*-regulatory effects across studies, populations and cell types, contrary to *trans*-effects, which may either be more cell/tissue-specific, may have smaller effect sizes or may be false positives. There was also no evidence of "master regulators" in this study. The authors demonstrated the utility of the eQTL approach in complex trait mapping, by identifying eSNPs which associate with HDL-cholesterol levels and expression levels of *VNN1*. They ultimately identified variants in this gene with functional consequences on transcription binding.

The first eQTL study to systematically compare two different tissues and pursue combined genetic analysis of expression levels and complex traits -in this case obesity-related phenotypes[39]- assessed 20,877 transcripts in the blood (IFB cohort; n = 1,002) and adipose tissue (IFA cohort; n = 673) of Icelandic families, genotyped for 1,732 microsatellite markers for linkage analysis and also for 317,503 SNPs for association analysis in a subset of 150 unrelated subjects. In this study, adipose tissue expression levels (63-72%) correlated better with obesity traits than do blood expression levels (3-9%), and this effect was more pronounced when the analysis was confined to the subset of subjects that overlapped in the blood and adipose tissue cohorts. Fifty-five percent of blood and 75% of adipose tissue transcripts were significantly heritable, with average heritability estimates of 30%, similar to the estimates from prior studies[29,32,37,38]. Variance components linkage analysis revealed 2,529 (12%) significant *cis*-eQTLs in blood, and 1,489 (7%) in adipose tissue, where *cis*-eQTL region corresponded to the microsatellite located nearest the expression probe of interest. Greater than 50% of the significant adipose tissue *cis*-eQTLs were also significant in blood. Expression traits with higher heritability in both tissues had greater reproducibility of eQTL signals. Thus, although there is significant overlap in genetic factors underlying gene expression in two different tissue types, expression levels from the clinically-relevant tissue appears to correlate better with clinical-phenotypes. There were many less significant *trans*-eQTLs and no evidence of "master regulators" above what would be expected by chance alone. There were 2,714 (12.9%) significant *cis*-SNPs in blood and 3,364 (16%) in adipose tissue, where *cis*-SNPs were defined as those residing within a 2 Mb window centered at the location of the probe corresponding to the transcript of interest.

This study, by Emilsson et al.[39], also characterized the transcriptional network by evaluating all pair-wise correlations in the most differentially expressed genes and generated a "connectivity map", which defined "connectivity" of a given gene as "the sum of its connection strengths with all other genes in the network". This approach identified a group of highly correlated gene-expressions (termed "network module") in human adipose tissue, which significantly overlapped with a "network module" in mouse adipose tissue. This module was found to be significantly enriched for genes in macrophage activation pathways. These genes also had *cis*-eSNPs which significantly influenced expression levels as well as obesity-related traits. These results collectively demonstrate that expression correlation networks combined with *cis*-eSNPs could potentially identify genes/pathways underlying complex clinical phenotypes.

Using similar approaches with Emilsson et al.[39], but human liver tissue from 427 unrelated subjects, Schadt et al.[40], performed an eGWAS using levels of 39,280 transcripts (34,266 genes) and 782,476 SNPs. At Bonferroni adjusted p < 0.05, there were 1,350 expression traits (1,273 genes, 3.7%) and at FDR <10%, 3,210 traits (3,043 genes; 8.8%) which had ≥1 significant *cis*-eSNP, explaining 2-90% of variation in expression levels. In contrast, significant *trans*-eQTLs were far fewer with only 242 traits (236 genes) or 491 traits (474 genes) showing significance after Bonferroni or FDR corrections, respectively. Comparison of their former blood and adipose tissue eQTLs[39] to the liver eQTLs determined 30% overlap of *cis*-eQTLs from each tissue with significant liver *cis*-eQTLs. By combining their liver eGWAS with publicly available disease GWAS data and transcriptional network approach[39], Schadt et al.[40] identified *SORT1 *and *CELSR2 *as candidate genes for coronary artery disease and LDL cholesterol levels, and *RPS26 *for Type 1 diabetes. These findings have implications for mapping those loci that influence disease risk by affecting gene expression levels. They demonstrate how the combined eQTL and disease mapping approaches can help distinguish the actual disease susceptibility gene in regions of LD, where SNPs in more than one gene may associate with disease risk, but only the disease-related transcript levels will associate with the eSNPs.

There are two published eGWAS utilizing transcript levels from human brain tissue[41,42]. The first study by Myers et al.[41] assessed neuropathologically "normal" cerebral cortical tissue from 193 subjects for 14,078 transcripts and 366,140 SNPs. They determined that 58% of the transcriptome had expression in ≥5% of control brains. Of these, 21% had significant associations with a *cis*- or *trans*-eSNP. Contrary to prior studies[34,35,37-40] performed in human LCL, lymphocyte, adipose and liver tissue, human brain eGWAS identified less significant *cis*-eSNPs (433 eSNPs for 99 transcripts) than *trans*-eSNPs (16,701 eSNPs for 2,876 transcripts). There was, nevertheless, enrichment of significant *cis*- vs. *trans*-associations over chance expectations at distances close to the transcripts and maximizing at distances <70 kb. Myers et al.[41] determined that *MAPT cis*-eSNP alleles that are on the major H1 haplotype are associated with higher MAPT transcript levels, in agreement with their prior study[43]. Compared to Cheung et al.[33], and Stranger et al.[44], there were only two common results (1 *cis *and 1 *trans*-association), in contrast to 30% overlap of *cis*-eQTLs between liver[40] and blood or adipose tissue[39] transcriptomes. These differences could arise from differences in platform and/or may suggest a more distinct genetic control for the brain transcriptome. Technical challenges arising from measuring levels of potentially degraded transcripts in post-mortem brain tissue[45], in comparison to other tissues that may have more well-preserved RNA, may also underlie some of these discrepancies.

In the follow-up brain eGWAS, Webster et al.[42], assessed 176 brain samples with AD neuropathology, and performed a joint evaluation with 188 control brains from their prior study[41]. The analyses were restricted to 8,650 transcripts out of 24,357 (35.5%), which were detected in >90% of the cases and controls. Similar to control brains, 58% of the transcriptome was detectable in ≥5% of AD brains. Assessment of technical and biological covariates revealed that hybridization date and APOE had the strongest influence and post-mortem interval had the least influence on brain expression levels. There were 1,829 significant *cis*-eSNPs (within gene ± 1 Mb of 3' or 5' end) in their combined sample and 656 significant *trans*-eSNPs. Twenty-seven percent of all eQTLs were found to have significant interaction term with diagnosis. Of the *cis*-eSNPs with significant diagnosis interaction, 37% were also found in just the control brains. Thus, a subset of *cis*-eSNPs that influence transcript levels differentially for AD subjects, also influence gene expression in control brains, suggesting that a portion of disease-relevant eSNPs may be captured in disease-free tissue and also that presence of other factors besides the disease-relevant eSNPs are likely necessary to predispose to AD. The authors did network analysis on transcripts with a significant eQTL and those that were differentially expressed between ADs and controls, but did not have a significant eQTL. This led to the identification of some transcript clusters that were significantly enriched for gene ontology groups; and "hub" genes with expression levels that correlate with many other transcripts. The authors highlighted their findings of significant *cis*-eSNPs that influence expression of *GSTO2*, but not *GSTO1*, which were previously implicated in AD risk[46,47], thereby implicating *GSTO2 *as the likely AD risk gene in this region.

Collective evidence from the eQTL studies discussed in this section lead to the following conclusions:

1) Transcriptome expression levels can be repliably and replicably detected in human cell lines and multiple types of tissues.

2) Genetic factors account for a substantial proportion (3-85% depending on study size, tissue source, expression platform) of the variation in human gene expression, with median heritability estimates of 20-30% in most studies. This is similar to the genetics of gene expression for other organisms.

3) Expression QTLs can be mapped by linkage or eGWAS approaches.

4) Most eQTLs appear to be *cis*-regulating, suggesting that *trans*-eQTLs may have smaller effect sizes.

5) Both SNPs and structural variations, namely CNVs, appear to underlie eQTL effects.

6) Many eQTLs are common across different ethnic groups, though there appear to be eQTLs that are unique to one ethnic group.

7) Many eQTLs are common across different tissue types, though there appear to be eQTLs that are unique to one tissue type.

8) "Gene expression endophenotype" approach is powerful and can detect hundreds to thousands of significant eQTLs with hundreds of subjects, unlike "complex disease mapping" approaches. Increasing sample size has the most impact on power for eQTL studies (vs. increasing SNP markers).

9) "Gene expression endophenotype" approach can be utilized in conjunction with mapping for complex diseases or other disease-related phenotypes to identify or confirm novel genes with functional eSNPs that confer disease risk. "Network" analysis of human eQTLs alone or in conjunction with those from other organisms can identify novel biological pathways that may be "disease-relevant".

10) "Disease-relevant" eQTLs are more likely to be captured in "disease-relevant" tissue (e.g. obesity-related eQTLs in adipose tissue)[39], although many such eQTLs may also be identified in other tissues. There appears to be substantial overlap in eQTLs detected from subjects with and without disease, although detection of some eQTLs may require assessment of "disease-relevant" tissue from subjects with disease.

## Gene Expression Endophenotypes in Neurodegenerative Diseases-Current Status

Gene expression endophenotypes have thus far been utilized in two types of studies in neurodegenerative diseases: "Candidate gene" studies, where influence of variants on expression levels of one or a few candidate genes are assessed; and "Expression profiling" studies, which compare levels of transcriptome expression in tissues from patients vs. controls in an effort to identify novel disease genes and pathways. This section provides examples of both types of studies to demonstrate the most common use of gene expression endophenotypes in neurodegenerative diseases to date. While not a comprehensive review on these studies, it will provide knowledge on these current approaches, including their uses and limitations.

### Gene Expression Endophenotypes in Neurodegenerative Diseases-"Candidate gene" approach

The initial studies utilizing the gene expression endophenotype in neurodegenerative diseases, have been conducted for known disease risk genes. A prime example of this is Apolipoprotein E gene (*APOE*), which is a well-established risk factor for AD, where the coding ApoEε4 polymorphism is associated with increased risk[48], but is neither sufficient nor necessary for its development (reviewed in[49]). Polymorphisms in the promoter region of *APOE*, which influence its expression, have received some attention as potential modifiers of AD risk that may be independent of ApoEε4 (reviewed in [50]). Although gene expression levels have not been directly used as endophenotypes in most of these studies, these polymorphisms were investigated because of their predicted influence on ApoE expression based on their location and *in-vitro *functional assays. One of the most well-studied of these polymorphisms is -491A/T, which was initially found to confer risk for AD in its -491AA homozygote form, even in subjects who lacked the risky ApoEε4 allele[51]. Functional transcriptional studies identified a stronger promoter activity for APOE -491A vs. -491T containing constructs[51]. ApoEε4 with the -491AA genotype had greater AD risk compared to those with one or no copies of the -491A allele, suggesting that both the isoform and level of expression of ApoE may be important in conferring disease risk and that ApoEε4 subjects with higher expression of this protein may be at highest risk[52]. While this notion is in agreement with findings from animal studies[53], the influence of ApoE promoter polymorphisms on brain ApoE levels have not been conclusive[50].

Another gene implicated in multiple neurodegenerative diseases, which was investigated with the expression endophenotype approach, is microtubule associated protein tau (*MAPT*). Missense and exon 10 splicing mutations in *MAPT *lead to frontotemporal dementia with parkinsonism linked to chromosome 17 (FTDP-17), whereas a region of LD within *MAPT*, known as the H1 haplotype is associated with increased risk of taupathies, namely, corticobasal degeneration (CBD) and progressive supranuclear palsy (PSP) compared with the (reviewed in [54]). Given the increase in *MAPT *exon 10-containing transcripts (also known as 4 repeat or 4R tau) in affected brain regions in PSP and CBD, Caffrey et al.[55,56] performed allele-specific gene expression studies in both human neuronal cell lines and brain tissue of *MAPT *H1/H2 heterozygous subjects. They determined that the risky *MAPT *H1 haplotype is associated with significantly higher *MAPT *exon 10-containing transcript expression without significant total *MAPT *levels compared to *MAPT *H2 haplotype. Myers et al. identified *MAPT *H1c as the sub-haplotype associated with an increase in both total and 4R tau levels in the human brain, with higher *in-vitro *transcriptional activity and with increased AD risk[43].

While the above examples demonstrate how the gene expression endophenotypes can be exploited to uncover the underlying biology of well-established genes in neurodegenerative diseases, our work utilizing the cerebellar expression levels of 12 late-onset AD (LOAD) candidate genes in 200 AD brains illustrate the use of this approach in identification of novel functional disease risk polymorphisms[12]. In this study, Zou et al. investigated association of 619 *cis*-SNPs with cerebellar expression levels of 12 LOAD candidate genes and identified three significant *cis*-SNPs in insulin-degrading enzyme (*IDE*). The top *cis*-SNP (rs7910977) reached genome-wide significance where the minor allele led to ~twofold increase in cerebellar IDE mRNA levels, reduced AD risk, and reduced plasma Aβ levels[57], which is biologically congruent. This *IDE cis*-SNP was in complete LD with an *IDE *SNP in a conserved region (rs6583817) and increased reporter gene expression in an *in-vitro *assay, providing additional evidence for a functional effect of this polymorphism on gene expression.

### Gene Expression Endophenotypes in Neurodegenerative Diseases-"Expression Profiling" approach

Large-scale comparisons of gene expression levels in subjects with disease vs. controls, known as "Expression Profiling" is one of the most commonly used approaches implementing the gene expression endophenotype. While potentially illuminating, this approach is vulnerable to technical confounders that may influence gene expression differentially in subjects with disease vs. controls, including tissue and RNA integrity[45]. To minimize these confounds, disease vs. control tissues need to be carefully matched and/or these variables need to be accounted for in the statistical analyses. Additional technical confounders to consider and control are microarray platform and quality and batch effects for experiments conducted on different dates[58]. There are important statistical considerations including variability in expression levels, which may lead to false positive findings especially for low expressing genes with high variability, as well as false negative results for those with small, but reproducible changes[58]. Perhaps the most important biological caveat is that, expression profiling design, especially if conducted in the disease-relevant tissue, does not distinguish expression changes that are a result of the disease process from those that are underlying causes of it. In this aspect, it is inferior to the eQTL approach, which can be designed to uncover the genetic factors underlying expression changes and disease risk. Despite these pitfalls, the potential utility of the expression profiling approach will be discussed in this section, highlighting the results from several expression profiling studies in neurodegenerative diseases.

In a hippocampal gene expression profiling study of 9 control and 22 AD subjects of various severity, determined by the bedside cognitive test, Mini Mental State Examination (MMSE)[59], Blalock et al.[60] correlated expression levels with both cognitive (MMSE) and neuropathology (neurofibrillary tangle) scores, in all subjects as well as the subset of nine controls or mild ADs with MMSE scores of 20-26 (collectively termed as the "Incipient ADs"). They identified upregulation of genes that pertain to transcription factor and tumor suppressor pathways among others. The small sample size and the inability to discriminate expression changes "due to" vs. "underlying" disease are the main concerns with this otherwise novel approach, which utilized three endophenotypes.

Bossers et al.[61] utilized a similar approach by generating expression profiles of 49 prefrontal cortex samples from subjects with different severity of underlying AD neuropathology detected by Braak staging for neurofibrillary tangles[62]. There were 1,071 transcripts (922) genes which showed significant changes in their expression levels with Braak stage. Some of these findings were validated with quantitative PCR (qPCR). There were clusters of genes which appeared to show concerted changes with advancing Braak stage, such as increasing early and then decreasing (UPDOWN clusters) or vice versa (DOWNUP clusters), where the biggest changes coincide with the appearance of amyloid plaques at Braak stage III. Functional annotation and pathway analysis of these clusters, revealed an enrichment for synaptic genes in the UPDOWN cluster and those involved in proliferation, differentiation and inflammation in the DOWNUP clusters. Importantly, the synaptic gene expression changes correlated with Aβ levels, which led to the conclusion that synaptic activity and Aβ production may be part of a feed-back loop that ultimately leads to AD. Given the appearance of changes even before significant neuropathology or clinical decline, the authors suggest that these findings are not a result of neuronal loss due to disease process.

While the previous examples are aimed at understanding the role of gene expression changes in the pathophysiology of a single neurodegenerative disease[60,61], Bronner et al.[63] performed expression profiling in the medial temporal cortex of 5 patients each from four distinct disease categories, namely, PSP, FTD, AD and PiD (Pick's disease) as well as a control group. Comparison of gene expression profiles between each disease group against controls identified a set of 166 transcript probes, which could discriminate PSP, FTD/PiD from controls and each other. The FTD and PiD groups have similar gene expression profiles. AD had the most similar profile to control group, in this small study. Although given its very limited sample size, this study should be considered as a pilot, it also demonstrates another potential application of gene expression profiling as a means to discriminate between neurodegenerative disorders at a molecular level.

Combining genetic association studies with gene expression profiling represents another paradigm utilizing this approach[64,65]. Taguchi et al. identified 35 genes that were significantly up- or down-regulated in the hippocampus of AD vs. control subjects, which they tested for genetic association with AD risk[64] in 376 AD patients vs. 376 controls. This study identified nine nominally significant AD risk associations, a higher significance rate than most such studies, with the *POU2F1 *association also reaching study-wide significance. Similarly, Chapuis et al.[65] performed case-control genetic association analysis for 82 genes that were found to be differentially expressed between 12 control and 9 AD brains, and determined nominally significant associations with AD risk for 17 genes. Of these, the association for *IL-33 *achieved study-wide significance, which was replicated in three additional series and was found to interact with ApoEε4. The rare allelic variants in *IL-33 *were associated with decreased risk of AD risk and reduced levels of cerebral amyloid angiopathy. Furthermore, overexpression of IL-33 was associated with decreased Aβ40 secretion *in-vitro*. Though replication studies are necessary, these studies illustrates the utility of gene expression profiling studies in identifying candidate disease genes, some of which may subsequently be shown to associate with both disease risk[64,65] and other disease-related phenotypes[65].

## Gene Expression Endophenotypes in Neurodegenerative Diseases-The Future

Available eQTL studies reviewed above illustrate the power of utilizing gene expression endophenotypes in conjunction with the disease phenotype, to identify novel genes, variants and pathways implicated in complex diseases. This approach, already applied to HDL-cholesterol levels[38] and obesity[39], can provide an alternative to the current genetic mapping approaches in neurodegenerative diseases. Distinct from candidate gene and expression profiling studies, eQTL investigations in neurodegenerative diseases will aim to identify genetic loci that influence both gene expression and disease risk at the genome-wide level.

The motivation to exploit the gene expression endophenotype as an alternative in the genetic mapping of neurodegenerative disease loci stems in part from the relative shortcomings of GWAS of complex diseases[9]. First, though multiple genetic loci have been identified for complex diseases through disease GWAS, these genetic variants fail to account for a substantial proportion of the underlying genetic risk[1,9]. To give AD as an example, there are 12 published LOAD GWAS[1,66], which led to the identification of a handful of genes that achieved genome-wide significance. These novel LOAD candidate loci collectively explain only a modest proportion of AD risk (reviewed in[1]), despite evidence that genetics account for ~80% of the risk for AD[67]. Gene expression endophenotypes appear to be more powerful than the disease phenotype, given the identification of hundreds to thousands of eQTLs in studies of only hundreds of subjects and may therefore identify genetic variants that may be missed by the classical disease mapping approach. Furthermore, they may allow for selecting a subset of the suggestive disease risk associations for further follow-up, by providing additional evidence for their functionality.

A relative concern with the eQTL approach is that since genetic studies may well be powered to detect eQTLs but underpowered to detect disease loci, complementary studies may be necessary to demonstrate that a particular eQTL also confers disease risk. Such studies may include concomitant mapping of other disease QTLs (e.g. serum/CSF biomarkers such as Aβ, neuropathology scores, cognitive measures); identification of eQTLs that are enriched for pathways previously implicated in disease; accepting less stringent significance for disease risk, but seeking replication in additional disease cohorts; functional studies with the identified genes to show their role in a disease-related *in-vitro *paradigm. It should also be noted that the eQTL approach can only capture those variants that confer disease risk via affecting gene expression and will miss coding changes that do not change transcript levels.

Another advantage of the eQTL approach is that the mechanism of action of the identified variants is already evident, which may allow for immediate downstream validation experiments such as measurement of protein levels in the brain or *in-vitro *transcriptional activity studies. Finally, combined eQTL and disease mapping approaches may identify the true disease-risk gene in regions of high LD spanning multiple genes, since the gene with the transcript that is influenced by the risk variant is most likely to be the disease-risk gene.

To achieve maximal benefit from the expression endophenotype approach in mapping novel neurodegenerative disease loci, attention should be given to technical aspects of the study design to minimize experimental confounds. These include choice and quality of tissue and transcriptome measuring platform, RNA integrity, statistical analysis to control for confounders and to detect significance[45,58], to name a few.

It should be mentioned that in addition to eSNPs and coding variants, epigenetic mechanisms are also recognized as an additional source of influence for risk of complex diseases such as cancer and AD[68,69]. Epigenetics comprise those reversible and dynamic mechanisms that influence gene expression, usually independent of DNA sequence, and include processes such as DNA methylation, histone acetylation and microRNAs[68,69]. Seminal work in DNA methylation profiles from monozygotic and dizygotic twins provided evidence for an important role of epigenetics in heritability that is also environmentally regulated[70]. Genome-wide epigenomic approach of investigating DNA-methylation differences in brains of subjects with schizophrenia and bipolar disorder identified epigenetic differences at numerous loci associated with psychosis, thereby providing evidence for the potential utility of this approach in CNS diseases[71]. A comprehensive review of epigenomic approaches in gene discovery for neurodegenerative diseases is beyond the scope of this review. Suffice it to say that the combined eQTL and disease GWAS approaches advocated in this review can be further enhanced by additional incorporation of the epigenomic approach as reviewed elsewhere[68,69].

## Concluding Remarks

Understanding the underlying genetic component of complex diseases, including Alzheimer's and other neurodegenerative diseases, has proven to be a challenge, despite the advances made mainly via GWAS of the dichotomous disease traits. Powerful approaches that constitute an alternative and are complementary to the current disease mapping algorithms are needed to overcome this challenge. Gene expression endophenotypes, which have a substantial genetic component, have already been used in mapping and functional validation of a few complex diseases and traits. While not all disease variants are expected to operate by changing transcript levels, it is expected that there will be many that confer disease risk by influencing gene expression. Such variants for neurodegenerative diseases may be captured by combining gene expression endophenotypes with existing disease GWAS to a) identify novel disease genes/pathways; b) validate suggestive findings from disease GWAS; c) elucidate the mechanism of action of newly discovered disease genes. Given their potential, gene expression endophenotypes are expected to be utilized in gene discovery for neurodegenerative diseases in the years to come.

## Abbreviations

AD: Alzheimer's disease; ALS: Amyotrophic lateral sclerosis; APOE: Apolipoprotein E; CBD: Corticobasal degeneration; CEPH: Centre d'Etude du polymorphisme humain; CNV: Copy number variant; eGWAS: Expression genome-wide association study; eQTL: Expression quantitative trait locus; FDR: False discovery rate; FTD: Frontotemporal dementia; FTDP-17: Frontotemporal dementia with parkinsonism linked to chromosome 17; GC: Genetic correlations; GO: Gene Ontology; GWAS: Genome-wide association study(ies); IDE: Insulin-degrading enzyme; Kb: Kilobase; KEGG: Kyoto Encyclopedia of Genes and Genomes; LCL: Lymphoblastoid cell line; LD: Linkage disequilibrium; LOAD: Late-onset Alzheimer's disease; MMSE: Mini Mental State Examination; MAPT: Microtubule associated protein tau; PC: Pearson's correlations; PD: Parkinson's disease; PiD: Pick's disease; PSP: Progressive supranuclear palsy; qPCR: Quantitative polymerase chain reaction; SNP: Single nucleotide polymorphism;

## Competing interests

The authors declare that they have no competing interests.

## Acknowledgements

**Funding: **R01032990 and P50AG016574 (PI: Ronald Petersen) to NET
